# The effectiveness of acupoint application of traditional Chinese medicine in treating primary dysmenorrhea: A protocol for meta-analysis and data mining

**DOI:** 10.1097/MD.0000000000026398

**Published:** 2021-06-18

**Authors:** Yanze Liu, Lin Yao, Bing Yan, Hailin Jiang, Jinying Zhao, Jiazhen Cao, Mengyuan Li, Xiaona Liu, Lijuan Ha, Li Tie, Chengyu Liu, Fuchun Wang

**Affiliations:** Changchun University of Chinese Medicine, No. 1035, Boshuo Rd, Jingyue Economic Development District, Changchun 130117, China.

**Keywords:** acupoint application, complementary and alternative therapy, data mining, primary dysmenorrhea, protocol, systematic review

## Abstract

**Background:**

Primary dysmenorrhea (PD) is a functional disease of the female reproductive system, which has adverse effects on patients’ physical and mental health and quality of life. At present, acupoint application of traditional Chinese medicine (TCM) as adjuvant therapy is undergoing clinical trials in different medical centers. However, there is no systematic review or meta-analysis to evaluate the efficacy of acupoint application of TCM in the treatment of PD. There is also a lack of systematic evaluation and analysis of acupoints and herbs.

**Methods:**

All randomized controlled trials related to acupoint catgut embedding therapy on PD will be searched in the following electronic databases: Cochrane Central Registry of controlled trials, PubMed, Wed of Science, EMBASE, Science Net, China Biomedical Literature Database, China Science Journal Database, China National Knowledge Infrastructure and Wan-Fang Database, from inception to May, 2021 were searched without language restrictions. The primary outcomes contain visual analog score, The Cox Menstrual Symptom Scale, while the secondary outcomes consist of adverse events and the recurrence rate. Two reviewers will independently perform data selection, data synthesis, and quality assessment. Data meeting the inclusion criteria will be extracted and analyzed by Revman v.5.3 software. Two reviewers will evaluate the study using the Cochrane collaborative bias risk tool. We will use the scoring method to assess the overall quality of the evidence supporting the main results. We will also use Spass software (version 19.0) for complex network analysis to explore the potential core prescription of acupoint application of traditional Chinese medicine in the treatment of PD.

**Results:**

This study will analyze the clinical effective rate, functional outcomes, quality of life, improvement of clinical symptoms of PD, and effective prescriptions of acupoint application for patients with PD.

**Conclusion:**

Our findings will provide evidence for the effectiveness and potential treatment prescriptions of acupoint application for patients with PD.

PROSPERO registration number: CRD 42021244357

## 1 Introduction

Primary dysmenorrhea (PD) is a kind of functional pain without organic causes,^[1]^ which is characterized by menstrual pain, mostly spasmodic, concentrated in the lower abdomen.^[2]^ Other symptoms include headache, weakness, dizziness, nausea, vomiting, diarrhea, and low back pain. This is a very common disease in young women.^[3]^ Parkinson disease is not only a painful experience but also affects women's psychological and emotional state.^[4]^

PD is not accompanied by obvious pelvic organic diseases. Age is an important factor in the occurrence of dysmenorrhea. Dysmenorrhea occurs rarely in the first few months of menarche and then increases rapidly.^[5]^ It reaches the peak at 16–18 years old (82%).^[6]^ It gradually decreases after 30–35 years old. It is stable at about 40% in the middle of childbearing age, and even lower at 50 years old. The results showed that the incidence of dysmenorrhea decreased from 72% to 67% by the age of 24, and the severity of dysmenorrhea also decreased. Childbirth is another important factor.^[7,8]^ The incidence and severity of dysmenorrhea in women with a history of full-term pregnancy and childbirth are significantly lower than those without a history of pregnancy and with pregnancy. In addition, women with early menarche or long menstrual period have severe dysmenorrhea, and the incidence of dysmenorrhea is significantly reduced in those taking contraceptives The mothers and sisters of dysmenorrhea patients often have dysmenorrhea.^[9]^ Dysmenorrhea is also related to mood, exercise, eating habits, environment, and other factors.^[10]^

Studies have shown that women with dysmenorrhea have high levels of prostaglandins, which are known to cause abdominal colic. Non-steroidal anti-inflammatory drugs (NSAIDs) can interfere by blocking the production of prostaglandins.^[11]^ Although nonsteroidal anti-inflammatory drugs can alleviate menstrual pain, about 18% of women with dysmenorrhea are unresponsive, leaving them and their physicians to pursue less well-studied strategies.^[12]^ In addition, epinephrine, contraceptives, progesterone, ibuprofen, analgesics, and other hormones are also frequently used in the clinic.^[13]^ These drugs have a quick effect, but the long-term effect is not good. Moreover, long-term use of such drugs may have side effects such as nausea, vomiting, dizziness, headache, etc.^[14]^

Acupoint application is a method of traditional Chinese medicine (TCM) with a long history.^[15,16]^ It uses TCM to externally apply on the skin and stimulate local acupoints to treat or prevent diseases.^[17,18]^ For PD, herbal patches are often applied to ST-36, RN4, RN6, EX-CA1, and SP-9 for 4 to 6 hours.

Acupoint application of TCM is a common method to treat chronic pain diseases. In recent years, its application in PD has been gradually popularized. Compared with other therapies, acupoint application has fewer side effects, faster effects, and higher patient compliance. However, the clinical efficacy of acupoint application of TCM in the treatment of PD and the possible therapeutic prescriptions are still unclear, which urges us to further explore its efficacy and effective prescriptions. Therefore, a systematic review of treatment results and prescriptions is helpful to better explain and apply this technique. This study aims to explore the existing evidence of the efficacy and safety of acupoint application of TCM in the treatment of PD, so as to help clinicians better apply it in clinical practice.

## 2 Methods

### 2.1 Study type

To evaluate the clinical effective rate, functional outcomes, quality of life, and side effects of acupoint application of TCM in the treatment of PD, we will collect randomized controlled trials (RCTs) for systematic review and meta-analysis. RCTs were conducted to compare the efficacy of acupoint herbal patch in the treatment of PD with no treatment, placebo, or conventional drugs (such as laxatives). All eligible trials will be included, including RCTs, observational studies, case series, and case–control studies. Animal experiments, reviews, case reports, qualitative studies, and studies with the repeated publication or incomplete data will be excluded.

### 2.2 Participants

Participants must meet the diagnostic criteria for PD. The diagnostic criteria refer to the clinical guidelines for PD formulated by the Canadian Obstetrics and Gynecology Association in 2017, and are not limited by gender, age, race, study area, and educational status. Similar symptoms due to specific pathological causes will be excluded, such as potential structural or metabolic diseases, as well as severe heart, liver, and kidney diseases.

### 2.3 Interventions

RCTs with acupoint application as the only intervention or the main part combined with other interventions (such as Chinese herbal medicine, western medicine, or other external treatment of TCM) will be included.

### 2.4 Comparisons

The intervention group was the acupoint application group, regardless of the treatment scheme, acupoint selection, and application time. The control group was given drug treatment, no treatment, Catgut Embedding at sham acupoints or placebo acupoints, acupuncture/electroacupuncture, and other interventions. Other interventions between the control group and the intervention group should be the same.

### 2.5 Outcome measures

The primary outcomes include:

1. Visual analog scale: the measurement of pain before and after the treatment period.
2. The Cox Menstrual Symptom Scale: The dysmenorrhea symptom score was performed, and the remission of dysmenorrhea symptoms before and after treatment was measured.

The secondary outcomes include:

1. Adverse events: fever, infection, bruising, numbness, local pain or ulcer, etc.
2. Recurrence rate.

### 2.6 Search strategy

An electronic search will be conducted. We will identify relevant studies from the Cochrane Central Register of Controlled Trials, PubMed, Embase, the Web of Science, the Chinese Biomedical Literature Database, the Chinese Scientific Journal Database, the Wan-Fang Database (Wanfang), and the China National Knowledge Infrastructure from their inception to May, 2021. The search term will consist of 3 parts: intervention method, disease, and study type: (“acupoint application” or “acupoint sticker” or “crude herb moxibustion” or “medicinal vesiculation” or “herbal patch” or “herbal plaster” or “acupoint patch” or “acupoint sticking” or “point application therapy” or “drug acupoint application” or “acupuncture point application therapies” or “plaster therapy” or “external application therapy” or “acupoint herbal patching”) and (“Primary dysmenorrhea” or “chronic Primary dysmenorrhea”) and (“randomized controlled trial” or “randomized” or “case-control studies” or “observational studies” or “case series” or “trial”), and (“blind”). The details of the PubMed Database search strategies are provided in Table 1. Similar but adaptive search strategies will be applied to other electronic databases. The language will be restricted to English and Chinese. Reference lists of relevant original studies will be screened to identify additional potential citations.

**Table 1:** The search strategy for PubMed database.

Serial number	Search items
#1	acupoint application [MeSH]
#2	acupoint sticker [MeSH]
#3	herbal patch [MeSH]
#4	herbal plaster [MeSH]
#5	acupoint patch [MeSH]
#6	acupoint sticking [MeSH]
#7	point application therapy [MeSH]
#8	drug acupoint application [MeSH]
#9	acupuncture point application therapies [MeSH]
#10	plaster therapy [MeSH]
#11	external application therapy [MeSH]
#12	acupoint herbal patching [MeSH]
#13	OR #1-#12
#14	dysmenorrhea [MeSH]
#15	menstrual cramps [MeSH]
#16	period pain [MeSH]
#17	menstrual pain [MeSH]
#18	painful menstruation [MeSH]
#19	menstrual colic [MeSH]
#20	primary dysmenorrhea [MeSH]
#21	primary dysmenorrheal [MeSH]
#22	essential dysmenorrhea [MeSH]
#23	primary painful menstruation [MeSH]
#24	primary pain [MeSH]
#25	primary menstruation ache [MeSH]
#26	OR #14-#25
#27	#13 AND #26

### 2.7 Study selection and data extraction

**Figure 1:**
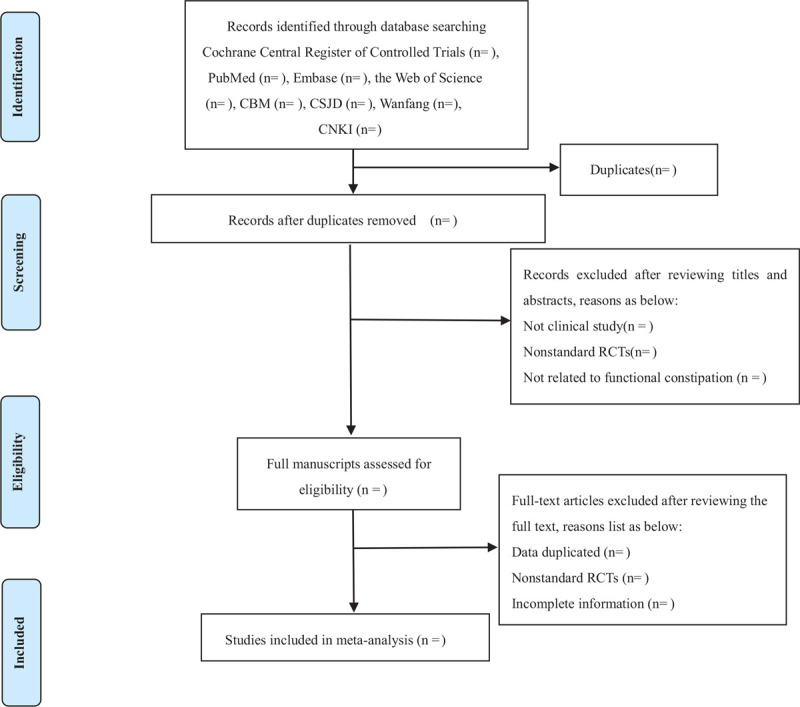
Flow chart of the search process.

This study will use Noteexpress 3.2.0 software (http://www.inoteexpress.com/aegean/) to exclude repetitive literature. The 2 reviewers (YL and BY) will independently evaluate the titles and abstracts of all citations found from the above search strategy. Copies of full-text articles can be used for potentially qualified studies. These reviewers will independently read the full-text articles that meet the standards; differences will be resolved by consensus through discussions with the third reviewer (JC). If the conclusion is still not reached, we will contact the author of the article. If the information in the article is missing, we will contact the study authors by email or telephone to obtain the missing data or other information. Otherwise, we will analyze the existing information and conduct sensitivity analysis to explore the potential impact of insufficient information on the results of a meta-analysis. Finally, the 2 authors (YL and LY) will extract data based on the recommendations of the Cochrane Handbook for a systematic review of interventions. The following data will be extracted: author, publication date, study country, study period, original inclusion criteria, randomness, sample size, baseline characteristics, intervention details, acupoints, herbal dosage and medication time, adverse events, follow-up period, recurrence rate, etc. The flow diagram of the study selection process is displayed in Figure 1.

### 2.8 Assessment of risk of bias

Two review authors (YL and HJ) will independently evaluate each included study and will follow the domain-based evaluation as developed by the Cochrane Handbook for Systematic Reviews of Interventions. They will assess the following domains: (1) selection bias (random sequence generation and allocation concealment), (2) performance bias and detection bias (blinding of participants, personnel, and outcome assessment), (3) detection bias (blinding of outcome assessment), (4) attrition bias (incomplete outcome data), (5) reporting bias (selective reporting), and (6) other bias (such as pre-sample size estimation, early stop of trial). Each domain will be divided into 3 categories: “low,” “high,” or “unclear.”

### 2.9 Data synthesis and analysis

Review Manager 5.3 software will be used to analyze the data.^[19]^ A meta-analysis using random or fixed-effects models will be conducted to summarize the data. Continuous data will be pooled and presented as mean differences or standardized mean differences with their 95% confidence interval (CI). Dichotomous data will be pooled and expressed as a risk ratio with their 95% CI. The following standards will be referred to: *I*^2^ values of 25% are considered low levels of heterogeneity, 50% indicated moderate levels, and 75% indicated high levels. Since low or moderate heterogeneity suggests little variability among these studies, the data will be analyzed in a fixed-effects model.^[20]^ When significant heterogeneity occurs among the studies (*P* < .05, *I*^2^ > 50%), a random-effect model will be performed to analyze the data.

### 2.10 Additional analyses

Subgroup analysis will be conducted to evaluate the specific influence of intervention type, age, course of the disease, and treatment duration on pooled results. In addition, sensitivity analysis will be performed to examine the robustness of the results by eliminating low-quality trials. Spass 19.0 software will be used for complex network analysis to explore the potential core prescription of acupoint application of TCM in the treatment of PD.

### 2.11 Reporting bias analysis

Reporting bias will be evaluated by visual inspection of Funnel plots. When asymmetry shows in the visual examination, the Egger method could be carried out for exploratory analysis.

### 2.12 Quality of evidence

We will assess the quality of evidence for the outcomes by Grading of Recommendations Assessment, Development, and Evaluation (GRADE) system. GRADE will be used to summarize the limitations in design, consistency, directness, precision, and publication bias. The quality of each evidence will be divided into 4 levels: high, medium, low, and very low. Disagreements will be resolved by consensus.

## 3 Discussion

PD is a spasmodic pain in the lower abdomen before and after menstruation or during menstruation, accompanied by dizziness, nausea, general discomfort, and even fainting due to severe pain. It has become an important public health problem and has a negative impact on women's health, social relations, work or school activities, and mental state, It is one of the most common gynecological diseases that affect women's quality of life and social activities. The prevalence of PD varies from 40% to 90% all over the world. In recent years, people's demand for improving PD pain is increasing. Studies have shown that most PD is caused by tissue ischemia and excessive muscle contraction. Excessive contraction of the spiral artery in premenstrual endometrium leads to endometrial ischemia, leading to endometrial shedding and spasmodic contraction of the myometrium, so as to push menstrual blood through the cervix and discharge from the vagina. There are many ways to treat PD, including western medicine and TCM. The treatment of PD in western medicine is mainly NSAIDs, slow-release analgesics, and oral contraceptives. The treatment of PD in TCM is mainly warming the liver, promoting blood circulation and removing blood stasis, soothing the liver and regulating qi, regulating menstruation, and relieving pain. Because some patients use drugs to intervene PD will have side effects or treatment effect is not obvious, so many patients with PD try to use complementary and alternative therapy, including acupoint herbal application (AHP). AHP is the TCM prescription fixed on the appropriate size of the patch, applied to specific acupoints, stimulates local skin, meridians, and acupoints, play a preventive and therapeutic effect on the disease.

Acupoint application is one of the common methods of TCM in the treatment of PD. Although acupoint application of TCM is often used in the clinical treatment of PD, a large number of related studies are also being carried out around the world. However, high-quality English reviews and meta-analyses on acupoint application for PD have not yet appeared. There is no systematic review on the efficacy and safety of acupoint application in the treatment of PD. Therefore, this paper aims to explore the effects of aupoint application of TCM on the clinical efficacy, functional outcome, quality of life, improvement of clinical symptoms, adverse events, and drug withdrawal events of patients with PD, so as to evaluate the efficacy, safety, reliability, and methodological deviation of acupoint application in the treatment of PD scientists and ordinary patients engaged in scientific research or the use of acupoint application therapy to provide a convincing basis.

## 4 Ethics and dissemination

Ethics approval is not required due to this work is carried out on published data. We aimed to explore the clinical effective rate, functional outcomes, quality of life, improvement of clinical symptoms of PD, as well as effective prescriptions of AHP for patients with PD. In the end, the results will be submitted to a peer-reviewed journal.

## Author contributions

**Conceptualization**: Yanze Liu, Jiazhen Cao.

**Data curation**: Mengyuan Li, Bing Yan, Jiazhen Cao.

**Formal analysis:** Yanze Liu, Xiaona Liu, Lijuan Ha.

**Funding acquisition:** Fuchun Wang.

**Investigation:** Lin Yao, Jiazhen Cao.

**Methodology:** Yanze Liu, Lin Yao.

**Project administration:** Jinying Zhao, Li Tie.

**Resources**: Yanze Liu, Bing Yan, Hailin Jiang, Chengyu Liu.

**Software**: Mengyuan Li, Chengyu Liu.

**Supervision**: Fuchun Wang.

**Validation**: Yanze Liu, Xiaona Liu.

**Visualization**: Fuchun Wang.

**Writing – original draft:** Yanze Liu, Lin Yao.

**Writing – review & editing:** Hailin Jiang, Li Tie, Fuchun Wang.
